# Reactivation of telomerase reverse transcriptase expression in cancer: the role of TERT promoter mutations

**DOI:** 10.3389/fcell.2023.1286683

**Published:** 2023-11-15

**Authors:** Maria Lina Tornesello, Andrea Cerasuolo, Noemy Starita, Sara Amiranda, Patrizia Bonelli, Franca Maria Tuccillo, Franco M. Buonaguro, Luigi Buonaguro, Anna Lucia Tornesello

**Affiliations:** ^1^ Molecular Biology and Viral Oncology Unit, Istituto Nazionale Tumori IRCCS Fondazione G. Pascale, Napoli, Italy; ^2^ Innovative Immunological Models Unit, Istituto Nazionale Tumori IRCCS Fondazione G. Pascale, Napoli, Italy

**Keywords:** telomerase, tumour, telomeres, TERT promoter mutations, TRF2, G-quadruplex, TERT inhibitors, TERT peptides

## Abstract

Telomerase activity and telomere elongation are essential conditions for the unlimited proliferation of neoplastic cells. Point mutations in the core promoter region of the telomerase reverse transcriptase (TERT) gene have been found to occur at high frequencies in several tumour types and considered a primary cause of telomerase reactivation in cancer cells. These mutations promote TERT gene expression by multiple mechanisms, including the generation of novel binding sites for nuclear transcription factors, displacement of negative regulators from DNA G-quadruplexes, recruitment of epigenetic activators and disruption of long-range interactions between TERT locus and telomeres. Furthermore, TERT promoter mutations cooperate with TPP1 promoter nucleotide changes to lengthen telomeres and with mutated BRAF and FGFR3 oncoproteins to enhance oncogenic signalling in cancer cells. TERT promoter mutations have been recognized as an early marker of tumour development or a major indicator of poor outcome and reduced patients survival in several cancer types. In this review, we summarize recent findings on the role of TERT promoter mutations, telomerase expression and telomeres elongation in cancer development, their clinical significance and therapeutic opportunities.

## 1 Introduction

**FIGURE 1:**
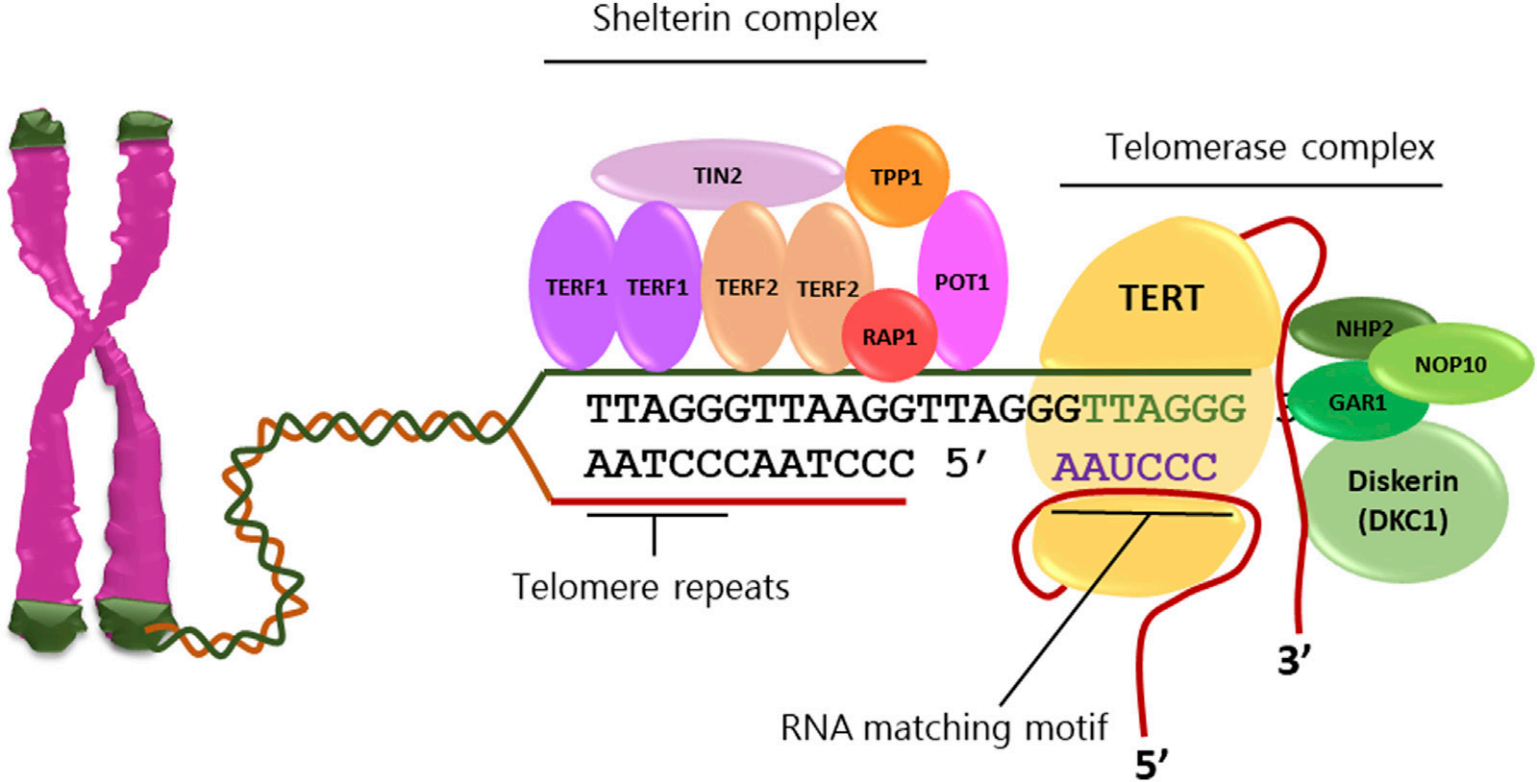
Telomeres are tandem repeats of nucleotide sequences (TTAGGG) protecting the chromosome ends from constitutive exposure to the DNA damage response, which are elongated by telomerase activity. Telomerase complex comprises the RNA template component (TERC), dyskerin, GAR1, NHP2, and NOP10 proteins as well as the reverse transcriptase holoenzyme TERT. The shelterin complex stabilizes telomeres through the direct binding of TERF1 and TERF2 homodimers as well as POT1 to telomeric sequences and stabilization by TIN2 and TPP1.

Cancer cells acquire replicative immortality through a multistep process involving progressive accumulation of genetic and epigenetic changes, escape from replicative senescence and neoplastic transformation (Hanahan and Weinberg, 2011). Telomerase activity, commonly low or undetectable in normal somatic cells, is frequently reactivated in cancer cells causing elongation of telomeres at chromosomes ends and loss of the Hayflick limit (Shay and Wright, 2000; Jafri et al., 2016). Telomerase is a ribonucleoprotein complex formed by the RNA template component (TERC) and a tetrad of proteins including dyskerin, GAR1, NHP2 and NOP10 as well as the reverse transcriptase holoenzyme TERT (Figure 1), (Smith et al., 2020). Furthermore, a protein complex formed by shelterin, POT1, RAP1, TIN2, TPP1, TRF1 and TRF2 contributes to the maintenance of telomeres by protecting terminal tandem repeats from DNA damage response (Smith et al., 2020). The level of TERT, its subcellular localization and assembly with telomeres, represent the limiting factors of telomerase activity, since all other associated components are constitutively expressed in both normal and neoplastic cells (Cristofari and Lingner, 2006).

Telomerase activity is upregulated in neoplastic cells through multiple mechanisms including chromosomal rearrangements, amplification of TERT gene, generation of TERT structural variants, disruption of telomere position effect and activating mutations in TERT promoter (Zhao et al., 2009; Peifer et al., 2015; Barthel et al., 2017). Epigenetic factors causing DNA methylation and histone acetylation also contribute to abnormal regulation of TERT transcription (Dogan and Forsyth, 2021). In addition, virus-related cancers, which commonly require constitutive expression of viral oncoproteins to sustain cell transformation, are characterized by high levels of telomerase activity. In particular, oncoviral proteins, such as high-risk human papillomavirus (HPV) E6, Epstein-Barr virus (EBV) LMP1, Kaposi’s sarcoma-associated herpesvirus (HHV-8) LANA, hepatitis B virus (HBV) HBx, hepatitis C virus (HCV) core protein and human T-cell leukemia virus-1 (HTLV-1) Tax protein, have been demonstrated to contribute to the transcriptional activation of the TERT gene by interacting with negative regulators of TERT transcription (Tornesello et al., 2022a).

The TERT gene is located approximately one megabase from the end of the short arm of chromosome 5, and its core promoter is delimited by nucleotides located at positions −330 upstream and +37 downstream of the TERT ATG start site (Cong et al., 1999; Takakura et al., 1999). TERT gene expression is tightly controlled by several regulatory factors that interact with binding sites in the core promoter region, such as GC-box and E-box motifs (Ramlee et al., 2016; Yuan et al., 2019). These factors include among others E2F1 (Crowe et al., 2001), Ets2 (Xu et al., 2008), c-Myc (Wu et al., 1999), NF-kB (Sheng et al., 2006), NFX1-123 (Gewin et al., 2004), p27KIP1 (Kanzawa et al., 2003), Sp1 (Oh et al., 2001), STAT3 (Wang et al., 2011) and TGFβ/Snail axis (Yoo et al., 2015), which in normal cells are subjected to spatial and temporal transcription changes during development, cell renewal and differentiation processes. However, most of these factors are aberrantly produced in tumour cells and are responsible for the activation of oncogenic pathways through the uncontrolled de-repression of several genes involved in cell growth and division, including TERT (Liu et al., 2016; Lambert et al., 2018; Bushweller, 2019).

Activating mutations in the proximal promoter of the TERT gene were first reported in cutaneous melanoma by two independent studies, and have subsequently be shown to occur more frequently than any other carcinogenic mutation in different tumour types (Horn et al., 2013; Huang et al., 2013; Huang et al., 2015a). These single nucleotide changes are G>A transitions, mostly arising at positions −124 and −146 upstream of TERT ATG start site, which create *de novo* binding motifs for E-twenty-six transformation-specific (ETS) transcription factors (Bell et al., 2015; Liu et al., 2018; Mancini et al., 2018). Moreover, whole genome sequencing of 326 cell lines derived from different cancer types showed that 60 lineages harboured mutated TERT promoters with a mutation rate similar to that of the respective primary tumours (Huang et al., 2015b). Importantly, all of them showed that mRNA monoallelic expression is specifically transcribed by the TERT allele with mutated promoter. Only cancer cell lines presenting TERT promoter mutations exhibited the H3K4me2/3 active chromatin marks and recruitment of the GABPA/B1 transcription factor to the cognate binding sites. Therefore, these findings suggest that single nucleotide changes in the TERT promoter cause major epigenetic changes and monoallelic expression (Vogelstein et al., 2013; Huang et al., 2015b; Stern et al., 2015; Akincilar et al., 2016; Xu et al., 2018; Takeda et al., 2020).

Recent findings have shown that hotspot changes in the TERT promoter cooperate with mutations in the regulatory regions of other telomerase-related genes, such as the G>A transition in the promoter of the ACD gene, which encodes the TPP1 protein of the shelterin complex (Chun-On et al., 2022). Furthermore, specific mutations in the TERT promoter have been found to frequently coexist with BRAF V600E variation in melanoma and observed to affect the disease-free survival of cancer patients (Del Bia et al., 2020).

In this review, we summarize diverse molecular mechanisms involved in telomerase activation in cancer cells harbouring TERT promoter mutations, their relationship with disease outcome in patients with different cancer types as well as the importance of anticancer therapies targeting telomerase.

## 2 TERT promoter mutations and activation of telomerase expression

Mutations consisting of G>A transitions at positions −57, −124 and −146 in the core promoter region of the TERT gene were first reported to occur in nearly 70% of melanoma as well as in different cell lines derived from several cancer types (Horn et al., 2013; Huang et al., 2013). All of these mutations were found to induce increased transcriptional activity in reporter gene assays. A further study, including 1,230 cases of 60 different tumour types, demonstrated that tumours can be classified into types with low (<15%) and high (≥15%) frequencies of TERT promoter mutations (Killela et al., 2013; Vinagre et al., 2013). Tumours harbouring low rates of mutations included colon cancer, breast cancer, and pancreatic cancer. On the other hand, high rates of TERT promoter mutations were identified in some tumour types including glioblastoma, bladder cancer, cutaneous melanoma, hepatocellular carcinoma and squamous cell carcinoma (SCC) of the skin and oral mucosa (Huang et al., 2015a).

More recently, a pan-cancer analysis of 10,336 cancer cases performed with a custom next-generation sequencing gene panel (MSK-IMPACT™, Memorial Sloan Kettering Cancer Center Integrated Mutation Profiling of Actionable Cancer Targets) provided relevant information regarding the distribution of TERT promoter variations in diverse tumour types and histological subtypes (Zehir et al., 2017). The data deposited in the cBioPortal database have been analysed for the distribution of TERT promoter mutations in cancer subtypes with at least 10 cases and results reported in Table 1. The high prevalence of TERT promoter mutations was confirmed in most tumour types such as glioma, bladder cancer, oral cavity carcinoma as well as melanoma and non-melanoma skin cancers (Zehir et al., 2017).

**TABLE 1:** Distribution of TERT promoter mutations in tumour types and histologic subtypes.

Tumour type/subtype^a^	Sample n	TERTp mut. n. (%)		TERTp C228T		TERTp C250T	
Skin Cancer							
Basal cell carcinoma	12	7	(58.3)	3	(25.0)	3	(25.0)
Squamous cell carcinoma	55	37	(67.3)	17	(30.9)	12	(21.8)
Merkel cell carcinoma	63	3	(4.8)	1	(1.6)	1	(1.6)
Melanoma	365	192	(52.6)	90	(24.7)	17	(4.7)
Brain tumour							
Anaplastic Oligoastrocytoma	16	4	(25.0)	3	(18.8)	1	(6.3)
Anaplastic Oligodendroglioma	43	41	(95.4)	33	(76.7)	8	(18.6)
Anaplastic Astrocytoma	86	33	(38.4)	22	(25.6)	11	(12.8)
Astrocitoma	35	7	(20.0)	6	(17.1)	1	(2.9)
Glioblastoma Multiforme	286	220	(76.9)	171	(59.8)	47	(16.4)
Gliosarcoma	10	9	(90.0)	8	(80.0)	1	(10.0)
High-Grade Glioma, NOS	20	6	(30.0)	6	(30.0)	-	
Oligodendroglioma	36	35	(97.2)	23	(63.9)	11	(30.6)
Thyroid Cancer							
Anaplastic Thyroid Cancer	33	27	(81.8)	24	(72.7)	3	(9.1)
Hurthle Cell Thyroid Cancer	23	13	(56.5)	13	(56.5)	2	(8.7)
Medullary Thyroid Cancer	17	-		-		-	
Papillary Thyroid Cancer	93	59	(63.4)	56	(60.2)	3	(3.2)
Poorly Differentiated Thyroid Cancer	60	34	(56.7)	31	(51.7)	3	(5.0)
Tumours of the reproductive system							
Clear Cell Ovarian Cancer	24	4	(16.7)	4	(16.7)	1	(4.2)
High-Grade Serous Ovarian Cancer	133	1	(0.8)	1	(0.8)	-	
Low-Grade Serous Ovarian Cancer	23	-		-		-	
Endometrial cancer	218	1	(0.5)	1	(0.5)	-	
Cervical cancer	50	3	(6.0)	1	(2.0)	2	(4.0)
Prostate Adenocarcinoma	698	2	(0.3)	2	(0.3)	-	
Urological tumour							
Bladder cancer	423	285	(67.4)	225	(53.2)	45	(10.6)
Renal Clear Cell Carcinoma	202	17	(8.4)	15	(7.4)	2	(1.0)
Papillary Renal Cell Carcinoma	33	11	(33.3)	10	(30.3)	-	
Chromophobe Renal Cell Carcinoma	33	1	(3.0)	1	(3.0)	-	
Head and neck cancer							
Head and Neck Squamous Cell Carcinoma	43	12	(27.9)	7	(16.3)	4	(9.3)
Larynx Squamous Cell Carcinoma	14	2	(14.3)	2	(14.3)	-	
Nasopharyngeal Carcinoma	13	-		-		-	
Oral Cavity Squamous Cell carcinoma	42	24	(57.1)	17	(40.5)	7	(16.7)
Oropharynx Squamous Cell Carcinoma	42	3	(7.1)	2	(4.8)	1	(2.4)
Salivary gland cancer	114	10	(8.8)	8	(7.0)	2	(1.8)
Soft tissue sarcoma							
Angiosarcoma	36	-		-		-	
Dedifferentiated Liposarcoma	45	-		-		-	
Desmoplastic Small-Round-Cell	22	3	(13.6)	3	(13.6)	-	
Leiomyosarcoma	41	-		-		-	
Myxoid/Round-Cell Liposarcoma	18	17	(94.4)	16	(88.9)	1	(5.6)
Rhabdomyosarcoma	11	-		-		-	
Sarcoma	32	4	(12.5)	4	(12.5)	-	
Solitary Fibrous Hemangiopericytoma	24	12	(50.0)	10	(41.7)	2	(8.3)
Synovial Sarcoma	34	-		-		-	
Undifferentiated Pleomorphic Sarcoma/Malignant Fibrous Histiocytoma/High-Grade Spindle Cell Sarcoma	59	10	(16.9)	8	(13.6)	2	(3.4)
Well-Differentiated Liposarcoma	18	-		-		-	
Digestive system tumour							
Cholangiocarcinoma	57	2	(3.5)	2	(3.5)	-	
Extrahepatic Cholangiocarcinoma	27	-		-		-	
Fibrolamellar Carcinoma	17	2	(11.8)	2	(11.8)	-	
Gallbladder Cancer	46	1	(2.2)	1	(2.2)	-	
Hepatocellular Carcinoma	85	37	(43.5)	35	(41.2)	-	
Intrahepatic Cholangiocarcinoma	115	5	(4.3)	5	(4.3)	-	

^a^ Only tumour subtypes with at least 10 individual cases analysed for TERT, promoter mutations have been included in the table.

### 2.1 Transcription factors and activation of mutant TERT promoter

**FIGURE 2:**
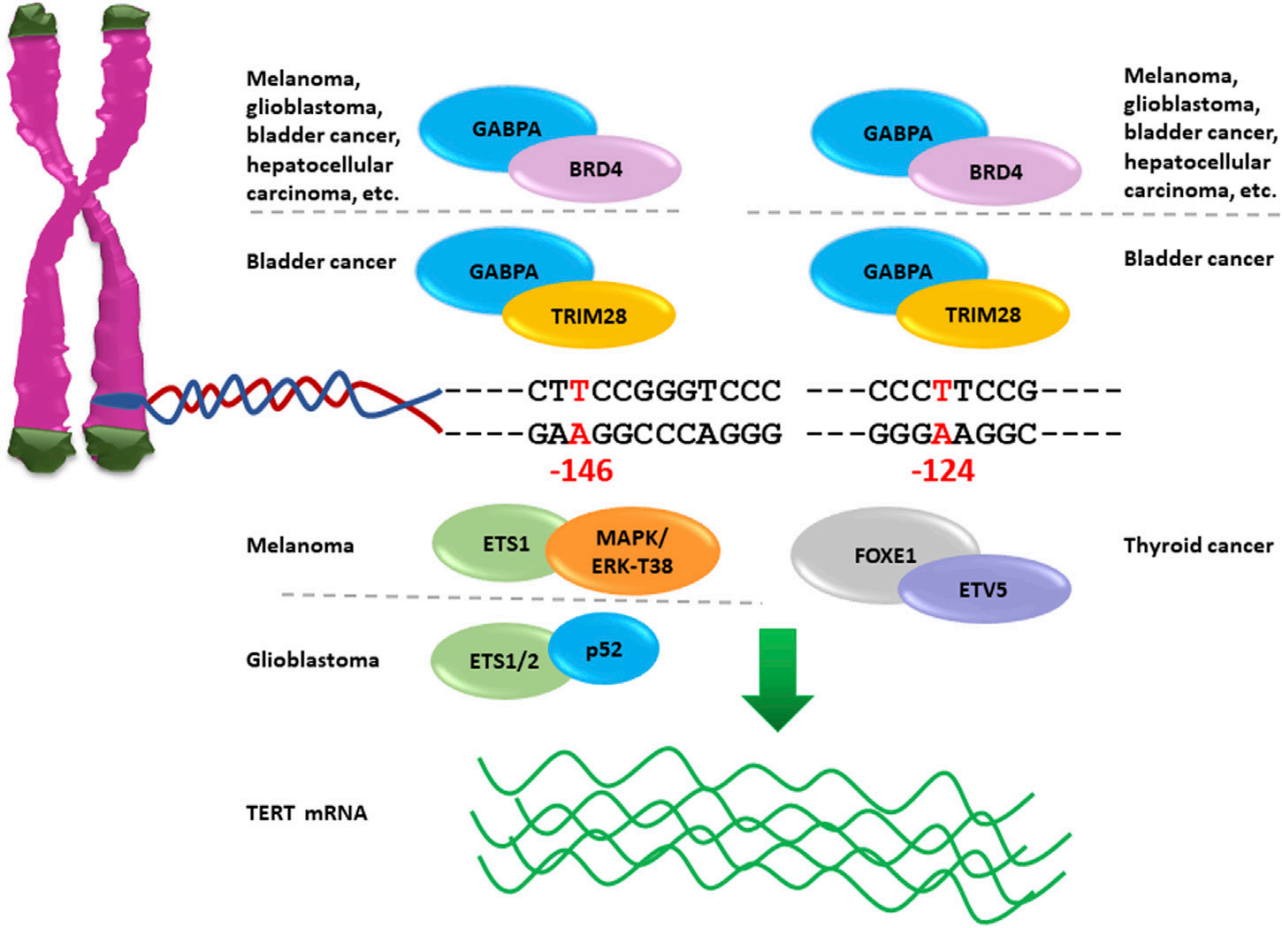
Nucleotide changes −124A and −146A in TERT promoter create novel binding sites for ETS factors reactivating telomerase expression. GABPA interacts with BRD4 and activates mutant TERT promoter −124A or −146A in several cancer types; NF-κB p52 recruits ETS1/2 to TERT promoter −146A causing reactivation of TERT gene expression in glioblastoma; the complex MAPK/ERK cause activation of TERT promoter −146A via the phosphorylation of ETS1 Thr38 amino acid in melanoma; FOXE1in combination with ETV5 activates TERT promoter −124A in thyroid cancer, and TRIM28 with GABPA is recruited on TERT promoter −124A and −146A to activate telomerase expression in bladder cancer.

TERT promoter mutations have been shown to activate telomerase expression through the creation of *de novo* 5′-GGAA-3′ binding motifs that are recognized by transcription factors of the E-twenty-six (ETS) family, including the GA-binding proteins alpha and beta (GABPA and GABPB), ETS1 and ETS Variant Transcription Factor 1 (ETV1) (Bell et al., 2015; Sizemore et al., 2017). In particular, the GABPA/B tetramer selectively binds and activates the mutant TERT promoter, thus inducing overexpression of the TERT gene in several tumour types, including glioblastoma, melanoma, hepatocellular carcinoma and bladder cancer (Figure 2), (Bell et al., 2015). TERT promoter mutations are mostly heterozygous, but only the mutant alleles recruit GABPA/B and exhibit epigenetic changes such as active histone marks (H3K4me2/3 and H3K9ac) as well as modifications in the chromatin architecture (Stern et al., 2015).

Three-dimensional (3D) organization of the genome represent an important mechanism by which regulatory elements control epigenetic modifications and gene expression (Dekker et al., 2013; Kim and Shay, 2018). The binding of GABPA dimers to mutant TERT promoters play a key mechanism in the formation of long-range TERT enhancer-promoter interactions and 3D chromatin reorganization. The transcription factor GABPA enables the interaction of mutant TERT promoter with a distal region (T-INT1) located 260 kb upstream TERT ATG start site and facilitates epigenetic changes that drive TERT gene transcription (Akincilar et al., 2016). Reversal of TERT promoter mutations, or deletion of T-INT1 region abrogates the GABPA binding to TERT promoter, depletes H3K4Me3 and H3K9Ac marks and leads to TERT gene repression. Therefore, 3D-chromatin organization is crucial factor for the activation of mutant TERT promoters (Akincilar et al., 2016).

Duplication of GABP binding sites in TERT promoter have been shown to cause the increase of telomerase expression similarly to the hot spot nucleotide changes (Barger et al., 2022). Duplications of a wild type sequence within the TERT promoter region, that include an ETS motif, have been identified in seven cancer types. All duplications had similar length ranging from 21 to 25 base pairs and insertion site, they recruit the GABP tetramer and activate the telomerase expression similarly to the single nucleotide mutations. Such findings suggest a strong selection for the recruitment of GABP to activate TERT expression (Barger et al., 2022).

Moreover, the complex formed by ETS1 with p52 NF-κB, whose expression is specifically controlled by non-canonical NF-κB signalling, has been shown to bind selectively the mutant TERT promoter −146A thus promoting telomerase expression in glioblastoma and melanoma (Li et al., 2015; Vallarelli et al., 2016). The ETS1 activity is dependent on the phosphorylation of Thr38 residue mediated by ERK, which is a component of the mitogen-activated protein kinase (MAPK) cascade permanently activated in BRAF or NRAS mutated tumours. Of note, BRAF variations coexist with TERT promoter mutations both in melanoma and in thyroid cancer (Dubbink et al., 2014; Liu et al., 2014; Xing et al., 2014; Shi et al., 2015; Jin et al., 2016). Importantly, melanoma cells harbouring the BRAFV600E mutation are characterized by constitutive activation of the RAS-ERK signalling pathway, which promotes recruitment of ERK2/GABP or ERK2/ETS1 complexes on the mutant TERT promoter, phosphorylation of SP1, shift in HDAC1 occupancy, acetylation of histones H3K9, and reactivation of telomerase (Shi et al., 2015).

Recent studies have identified activating mutations in the promoter region of the ACD gene co-occurring with TERT promoter mutations in 5% of cutaneous melanoma (Chun-On et al., 2022). The ACD gene generates two independent transcripts encoding a long and a short form of tripeptidyl peptidase 1 (TPP1), named TPP1-L and TPP1-S, respectively, which are involved in telomerase recruitment to telomeres and DNA synthesis (Grill et al., 2019). The TPP1-L isoform differs from TPP1-S by an arginine-rich region of 86 amino acids at its N-terminus and both retain the ability to recruit telomerase, but only TPP1-L inhibits telomerase function while TPP1-S facilitates efficient telomere synthesis. Mutations in the ACD gene are localized at nucleotide positions such that they cause amino acid changes in TPP1-L and at the same time generate new binding sites for ETS transcription factors, such as ETV5, ETV4 or ETS1, in the region functioning as promoter for the expression of TPP1-S (Chun-On et al., 2022). Importantly, such mutations drive the overexpression of TPP1-S, which in the presence of mutant TERT promoter and increased TERT levels lead to synergistic telomere lengthening and cooperate in the indefinite proliferation of melanoma cells (Chun-On et al., 2022).

In papillary thyroid cancers the ETS variant 5 (ETV5) is abundantly expressed and able to activate mutant TERT promoter in GABP-negative thyroid cancer cells (Chun-On et al., 2022). In addition, the ETV5 has been shown to interact with FOXE1, which is a thyroid-specific transcription factor crucial for thyroid development and differentiation, and to bind the mutant TERT promoter −124A causing the reactivation of telomerase expression (Bullock et al., 2019). Moreover, the co-existence of BRAFV600E and TERT promoter mutations in papillary thyroid cancer has been shown to have a synergistic effect on poor clinical outcome, probably due to the BRAF-dependent upregulation of several ETS transcription factors, especially ETV1, ETV4 and ETV5, causing telomerase over-expression associated with increased cancer invasiveness and progression (Song et al., 2019a).

In bladder cancer, GABPA is involved in the recruitment of two transcriptional co-regulators of telomerase expression, namely, TRIM28 (tripartite motif containing 28) and TRIM24, to the mutant TERT promoter −124A (Agarwal et al., 2021). The transcription factor TRIM28 is an activator while TRIM24 is a repressor of TERT expression. Hyperactivation of TERT promoter −124A in bladder cancer cells is caused by phosphorylation of TRIM28, mediated by the mTORC1 (mammalian target of rapamycin complex 1), which abrogates the TRIM28/TRIM24 interaction leading to displacement of the repressor and causing significant upregulation of TERT expression (Agarwal et al., 2021).

### 2.2 Telomere proteins and activation of mutant TERT promoter

Proteins of the shelterin complex, besides protecting telomeres from unwanted DNA repair processes, have a major role in the formation of loops and regulation of telomerase expression. Indeed, telomeric factors such as TRF1, TRF2 and RAP1 have the ability to interact with non-telomeric binding sites, such as TERT promoter sequences (Mukherjee et al., 2019). Recent studies have demonstrated that TRF2 binds the TERT promoter G-quadruplex and recruits the polycomb-repressor EZH2/PRC2 complex causing H3K27 trimethylation and TERT promoter repression both in cancer and normal cells (Sharma et al., 2021). However, mutations in the TERT promoter cause destabilization of G-quadruplex structures and TRF2 displacement resulting in telomerase overexpression in glioblastoma multiforme cells. These results showed that the telomeric factor TRF2 is a negative regulator of telomerase activity, but its function is hampered by the presence of mutations in the TERT promoter region.

## 3 Temporal and spatial acquisition of TERT promoter mutations

Somatic mutations in the TERT promoter have been identified with variable frequencies in numerous cancer types (Sharma and Chowdhury, 2022). Highest rate of mutations have been detected in several tumours including among others glioma (>70%), melanoma and non-melanoma skin cancers (>70%), hepatocellular carcinoma (>60%), bladder cancer (>60%) as well as squamous cell carcinoma of the penis (>50%), conjunctiva (>45%) and oral cavity (>30%), (Huang et al., 2015a; Pezzuto et al., 2017; Annu et al., 2018; Starita et al., 2018; Giunco et al., 2021; Starita et al., 2022). Studies on the clonality of mutations in the TERT promoter have contributed to define their temporal appearance and the oncogenic consequences. Indeed, these mutations have been defined as early events in some tumours and late events in others, consistent with the possibility that they play multiple oncogenic roles in diverse cancer types (Lorbeer and Hockemeyer, 2020).

In glioblastoma, TERT promoter mutations are considered early genetic events of tumour development. Lee et al. (2018) by performing a comparison study on the presence of TERT promoter mutations in brain tissues from matched tumour-free sub-ventricular zone, tumour and normal tissue, identified TERT promoter mutations and other oncogenic mutations in non-tumour tissues. Single-cell sequencing of a glioblastoma and its single-cell-derived clonal cell populations demonstrated that all clones had both private mutations as well as shared mutations with the correspondent tumour-free sub-ventricular zone, according to the model of progressive accumulation of driver mutations in cancer cells.

Benign melanocytic nevi harbour trunk nucleotide changes, such as BRAF and NRAS mutations, before progressing to melanoma and accumulate nucleotide changes in TERT promoter at the transition from benign nevus to melanoma *in situ* (Damsky and Bosenberg, 2017; Colebatch et al., 2022). Recently, Ciba et al. reported that TERT promoter mutations elicit low levels of telomerase expression in the first stage of melanoma development, which correlate with cell proliferation and healing of short telomeres but not with telomeres elongation (Chiba et al., 2017). The continuous cell division and critically short telomeres promote the transition to a second stage of neoplastic transformation involving genomic instability, progressive increase in telomerase levels and overgrowth of transformed melanoma clones (Chiba et al., 2017). The coexistence of mutations in BRAF and NRAS genes as well as in TERT promoter are associated with poor disease-free survival in melanoma patients (Nagore et al., 2016). Particularly, nucleotide changes at positions −138_139 CC>TT in TERT promoter have been shown to correlate with poorer clinical outcome in stage I and stage II melanoma patients, mainly in association with BRAF and NRAS mutations (Andrés-Lencina et al., 2019).

In primary hepatocellular carcinoma, the TERT promoter mutation −124A is the most frequent genetic alteration in HCV-related and metabolic-related tumours and represents approximately 95% of all TERT promoter variations (Müller et al., 2020). Nucleotide changes in the TERT promoter are considered trunk mutations in liver carcinogenesis, being identified in a consistent fraction of dysplastic nodules and at increased frequency in hepatocellular carcinoma (Nault et al., 2013; Totoki et al., 2014; Takeda et al., 2020; Fredriksson et al., 2014; Nault et al., 2014). In addition, the genetic landscape of liver cancer, characterized by nodule-in-nodule feature for the presence of the inner hypervascular tissue surrounded by hypovascular transformed hepatocytes, showed that the two components shared many clonal genetic events, including TERT promoter mutations (Takeda et al., 2020). Moreover, Ningarhari et al. (2021) reported that nucleotide variations in the TERT promoter were more frequent in the subtype of hepatocellular carcinoma characterized by short telomeres, well-moderate differentiation, and low levels of alpha-fetoprotein, rare vascular invasion, chromosomal stability and lower aggressiveness compared to non-mutated tumours. Detection of TERT promoter mutations in circulating tumour DNA of patients with hepatocellular carcinoma is associated with shorter survival and proposed as useful biomarker of poor prognosis in patients with advanced liver cancers (Pezzuto et al., 2018; Ge et al., 2021; Hirai et al., 2021).

Recurrent mutations in the TERT promoter have been detected in over 60% of urothelial bladder cancers and represent the most frequent molecular alterations in all grades and stages of urothelial cancer (Günes et al., 2018). Such mutations represent early events in the transformation process since they are detected in the urinary exfoliate cells decades before the diagnosis of bladder cancer. Indeed, in the Golestan cohort followed for up to 14 years, Hosen et al. identified the presence of TERT promoter mutations in urine samples of 47% asymptomatic individuals, who subsequently developed primary bladder cancer, and in none of matched controls who did not develop bladder cancer (Hosen et al., 2020). Nucleotide changes in TERT promoter were detectable up to 10 years prior to bladder cancer diagnosis. Then, identification of TERT promoter mutations in urinary exfoliated cells has emerged as a promising biomarker for early diagnosis of bladder cancer.

Squamous cell carcinoma of the conjunctiva is a common cancer in equatorial Africa mainly associated with exposure to solar ultraviolet and infection with human immunodeficiency virus (HIV). The frequency of TERT promoter mutations in intraepithelial neoplasia grade 1-3 (CIN1-3) and squamous cell carcinoma of the conjunctiva was significantly higher in HIV-positive (between 32% and 46%) compared to HIV-negative patients (between 13% and 22%) (Starita et al., 2018). These findings showed that TERT promoter mutations are early events in conjunctival neoplasia and that the immunosuppression caused by HIV infection strongly contribute to the accumulation of UV-related mutations in conjunctival lesions (Starita et al., 2018).

Variable frequencies of TERT promoter mutations have been detected in diverse tumour types of the lower genital tract, associated or not with human papillomavirus infection. Two independent studies performed in India and in Italy showed that 20% of cervical squamous cell carcinoma harboured TERT promoter mutations independently from the HPV status (Vinothkumar et al., 2016; Annu et al., 2018). The consistent association of TERT promoter mutations with cervical carcinoma is also supported by the presence of the G to A transition at position −146 in the HPV16-positive SiHa cell line, which is derived from a squamous cell carcinoma of the cervix (Annunziata et al., 2018). Notably, TERT mRNA expression levels were much higher in SiHa cells than in squamous cell carcinoma-derived CaSki and C-4I cells, as well as adenocarcinoma-derived HeLa cells, despite comparable expression levels of HPV E6. These data demonstrate that TERT promoter mutations have a stronger effect than HPV E6 transactivation on TERT gene expression. However, the rarity of recurrent TERT promoter mutations in CIN1-3 and adenocarcinoma of the cervix suggested that this is a late event in the development of cervical cancer.

Squamous cell carcinoma of the vulva originates from two different etiopathogenic pathways, one associated with human papillomavirus (HPV) infection and the other in the setting of longstanding dermatosis, such as lichen sclerosus (van der Avoort et al., 2006; Del Pino et al., 2013). Overall, TERT promoter mutations are highly frequent in the carcinoma of the vulva (>50%) particularly in HPV-negative tumours were the mutation rate exceeds 90% and coexists with other genetic alterations such as mutations in TP53 (87%), alteration in CDKN2A (67%), and mutations in NOTCH1 and FAT1 (47% each) (Salama et al., 2022). It remain to be clarified whether TERT promoter mutations are early or late events in HPV-independent squamous cell carcinoma of the vulva.

Similarly to vulvar cancer, penile squamous cell carcinoma either develop through an HPV associated pathway or through other oncogenic mechanisms independent from the HPV infection (Iorga et al., 2020). The search for TERT promoter mutations in penile carcinoma showed an overall prevalence above 50%, which was significantly higher in HPV negative cases (>65%) (Kim et al., 2021; Starita et al., 2022). Mutations in TERT promoter co-exited with nucleotide changes in PIK3CA gene in several cases. However, the frequency of mutant alleles showed discordant values indicating that PIK3CA mutations appear in subclones of TERT promoter mutated cells (Starita et al., 2022). Thereby, it is possible to conclude that TERT promoter mutations in penile carcinoma precedes the occurrence of other oncogenic mutations.

Squamous cell carcinoma of head and neck comprises a heterogeneous group of tumours mainly localized in the oral cavity, larynx, oropharynx, hypopharynx and nasopharynx. According to a recent systematic review and meta-analysis including 1830 patients, the overall prevalence of TERT promoter mutations in head and neck cancer was 21% with significant differences according to cancer site (Boscolo-Rizzo et al., 2023). Indeed, TERT promoter mutations are frequently detected in tumours of the oral cavity (47%) and larynx/hypopharynx (12%), while they are rare in squamous cell carcinoma of the oropharynx (1%). Notably, TERT promoter mutation −124A is the most frequent mutation and is associated with higher risk of disease progression and death while the mutation −146A has no significant correlation with disease outcome. The search for the mutation −124A should be considered as a promising diagnostic and prognostic biomarker in patients with oral squamous cell carcinoma.

## 4 Therapeutic opportunities

Selective reactivation of the telomerase in the large majority of tumours, while remaining inhibited in normal cells, provides the opportunity to develop therapeutic approaches inhibiting telomerase activity (Arndt and MacKenzie, 2016). Several strategies have been devised to target different components of the telomerase molecular machinery such as TERT mRNA, TERT holoenzyme, components of the telomerase complex, TERT promoter, nuclear factors regulating TERT expression as well as mutant TERT promoters (Tornesello et al., 2022b). Most telomerase inhibitors might be effective to treat several cancer types, such as prostate, lung, breast, colorectal, and haematological malignancies, which rarely harbour TERT promoter mutations but display strong telomerase activity mainly depending on chromosomal rearrangements and TERT gene amplification (Dratwa et al., 2020). In addition, new compounds that selectively stabilize the G-quadruplex in mutant TERT promoters and inhibit telomerase expression have been evaluated in preclinical studies for their inhibitory effect on telomerase and killing of cancer cells (reviewed in (Song et al., 2019b; Tornesello et al., 2022b)).

Immunotherapy targeting telomerase also showed promising results in all cancer types with high telomerase activity (Mizukoshi and Kaneko, 2019). Specifically, the GV1001 peptide containing a 16 amino-acid (616–626; EARPALLTSRLRFIPK), located within the reverse transcriptase domain of the telomerase holoenzyme, was originally developed as an anti-cancer vaccine to treat several cancer types (Negrini et al., 2020). A Phase I/II study evaluating the safety, tolerability and immunogenicity in patients with pancreatic cancer demonstrated the induction of an immune response that correlated with prolonged survival (Bernhardt et al., 2006). The GV1001 peptide showed cell penetrating (CPP) properties which enabled the cytosolic delivery of macromolecules such as proteins, DNA and siRNA via extracellular heat shock protein 90 (eHSP90) and 70 (eHSP70) complexes (Kim et al., 2016). The CPP properties may contribute to enhance its anti-cancer immune response compared to other telomerase peptide-based vaccines. Importantly, the GV1001 CPP, besides eliciting immune response against telomerase, has an antiviral effect against hepatitis C virus (HCV) and human immunodeficiency virus type 1 (HIV-1) as well as inhibits HBV replication and hepatitis B surface antigen (HBsAg) secretion in a dose-dependent manner (Choi et al., 2020). A recent study showed the anti-cancer effect of GV1001 in non-small cell lung cancer cells consisting in the suppression of cell proliferation and invasion as well as inhibition of VEGF expression (Kim et al., 2022). Therefore, GV1001 has also an inhibitory effect on tumour-associated angiogenesis.

Other TERT-derived synthetic peptides, that are presented by the major histocompatibility complex (MHC) and induce specific CD8^+^ cytotoxic T lymphocytes (CTL), include the peptide I540 (540-548; ILAKFLHWL), that is the first TERT immunogenic peptide identified as an HLA-A*0201-restricted T-cell epitope and has been evaluated in phase III clinical trials for melanoma treatment (Li et al., 2007; Wenandy et al., 2008; Liu et al., 2010). In addition, TERT 988 (988–997; DLQVNSLQTV) has been shown to stimulate CTL specifically against lysed tumour cells of various histological types expressing HER-2/neu or TERT (Scardino et al., 2002). Peptide K973 (973-981; KLFGVLRLK) was used to generate specific CD8^+^ CTLs from HLA-A3+ cancer patients and healthy individuals. These CTLs were able to lyse diverse HLA-A3 tumour cell types expressing telomerase (Vonderheide et al., 2001). TERT 572 (572-580; RLFFYRKSV) is a vaccine containing the adjuvant Montanide ISA51 and has been evaluated in patients with chemotherapy-resistant solid tumors. Two doses of vaccine induced the development of TERT 572Y -specific CD8^+^ in more than 90% of the vaccinated patients (Mavroudis et al., 2006). UV1 peptide vaccine, which consists of three peptides [(peptide 725; TERT 691-705 (RTFVLRVRAQDPPPE), peptide 719-20; TERT 660-689 (ALFSVLNYERARRPGLLGASVLGLDDIHRA), peptide 728; TERT 651-665 (AERLTSRVKALFSVL)] and GM-CSF as adjuvant, has been used in combination with androgen deprivation therapy and radiotherapy to treat patients with androgene-sensitive metastatic prostate cancer in phase I/II trial (NCT01784913). Long-term monitoring showed that some patients maintained high immune responses without relaps (Lilleby et al., 2023). UV1 peptide vaccination was also evaluated in patients with non-small cell lung cancer (NSCLC) and found to induce specific T-cell responses in the 67% of patients non-HLA-typed with stage III/IV NSCLC and with no evidence of progression after prior treatments (Brunsvig et al., 2020).

## 5 Conclusion

The crucial role of telomerase activity and telomere maintenance in cell transformation and development of cancers has known since decades. Mechanisms of telomerase reactivation in cancer cells include complex molecular changes such as chromosomal translocations, TERT gene amplifications, TERT structural variants, transcription binding factors, TERT promoter mutations and spatial chromatin arrangements. Most of these genetic and epigenetic alterations drive strong telomerase activity in a manner that is specific to cancer histotypes. Paradigmatic is the observation that a subgroup of high-risk neuroblastoma patients having mostly unfavourable outcome were characterized by TERT rearrangements or by MYCN amplified tumours, both inducing massive transcriptional upregulation of the TERT gene (Peifer et al., 2015). Similarly, increased TERT gene copy number and upregulation of telomerase have been identified in breast cancer, thyroid carcinoma, and lung adenocarcinoma in association with poor prognosis (Gay-Bellile et al., 2017). Targeting telomerase, by using direct telomerase inhibitors and immunotherapies, might be effective in all these cancers expressing high levels of TERT gene.

Knowing the temporal occurrence of genetic and epigenetic changes in TERT locus during cancer growth is very important for the development of diagnostic and prognostic markers in diverse tumour types. The cancer driver effect of TERT promoter mutations in tumour development has been shown by a proof-of-concept study showing that correction of the −124A mutation to the wild type −124G blocked the binding of ETS transcription factors to the TERT promoter, reduced TERT gene transcription and telomerase expression, and induced senescence and proliferative arrest of glioblastoma cells (Li et al., 2020). Assessment of the mutated TERT promoter in fluid matrices of human body has already demonstrated its value for the monitoring of tumour diagnosis and recurrence.

In normal cells, TERT promoter is tightly controlled by numerous transcription factors, which bind to regulatory elements and control the silencing in somatic cells and the reactivation in stem cells. These factors include among others activators of TERT transcription, such as the oncogene c-MYC, Sp1, NF-κB, STAT family of proteins, AP-2, GSC, as well as repressors that downregulate TERT transcription, such as MAD1/2, p53, WT1, CTCF, and MZF-2 (Dratwa et al., 2020). The frequent overexpression of transcriptional activators in human cancers, and mutations of repressors (i.e., p53) lead to the enhanced expression of TERT gene and disruption of the fine tuning of telomerase function. As an example, small molecules that cause disruption of Myc/Max heterodimerization on TERT promoter and proteasome-mediated degradation of Myc have been shown to suppress cell proliferation in diverse cancer cell lines (Choi et al., 2017; Han et al., 2019).

Mutations in the core promoter of the TERT gene are exceptionally high in many cancer types and may represent specific targets for new therapies. Such mutations abrogate the repressive function of G4-structures in the TERT promoter by inducing conformational changes (Palumbo et al., 2009; Chaire et al., 2014; Song et al., 2019b). Recently, the screening of chemical libraries allowed to identify small molecules able to bind specifically TERT promoter, to redirect the misfolding of mutant promoter G-quadruplexes and to re-establish the silencing of TERT gene (Song et al., 2019b). Furthermore, the tetrameric complex GABPA/GABPB plays an important role in the regulation of TERT transcription since it selectively binds and activates the mutant but not the wild-type TERT promoter in glioblastoma (Bell et al., 2015). A strong inducer of GABPB expression is FOS and T-5224, which is a new small molecule inhibitor of FOS, selectively suppress TERT expression in thyroid cancer cells carrying TERT promoter mutations but not in wild-type TERT cells (Liu et al., 2021). Therapeutic strategies specifically directed to TERT promoter mutations will likely have an important clinical impact in many types of human cancers characterized by such mutations.

## Figures and Tables

**Table:** 

**ACD (TPP1)**	Adrenocortical dysplasia protein homolog
**AP-2**	Family of transcription factors
**BRAF**	B-Raf proto-oncogene, serine/threonine kinase
**c-Myc**	MYC proto-oncogene
**CDKN2A**	Cyclin dependent kinase inhibitor 2A
**CTCF**	CCCTC-binding factor
**E2F1**	E2F Transcription Factor 1
**E6**	Human papillomavirus early protein 6
**ERK2**	Extracellular signal-regulated kinase 2
**ETS**	E-twenty-six transformation-specific
**ETS1**	ETS proto-oncogene 1, transcription factor
**ETS2**	ETS proto-oncogene 2, transcription factor
**ETV1**	ETS variant transcription factor 1
**ETV5**	ETS variant transcription factor 5
**EZH2**	Enhancer of zeste 2 polycomb repressive complex 2 subunit
**FAT1**	FAT Atypical Cadherin 1
**FGFR3**	Fibroblast growth factor receptor 3
**FOS**	Fos Proto-Oncogene, FOXE1, Forkhead box protein E1
**GABPA**	GA binding protein transcription factor subunit alpha
**GABPB1**	GA binding protein transcription factor subunit beta 1
**GABPB2**	GA binding protein transcription factor subunit beta 2
**GSC**	Homeobox protein goosecoid
**H3K4me2/3**	Demethylation or trimethylation of lysine 4 on histone H3 protein
**H3K9ac**	Acetylation of lysine 9 on histone H3 protein
**HBx**	Hepatitis B viral protein x
**HDAC1**	Histone Deacetylase 1
**HPV**	Human papillomavirus
**LANA**	Latency-associated nuclear antigen
**LMP1**	Latent membrane protein 1
**MAD1/2**	Mitosis arrest deficiency 1
**MAPK**	Mitogen-activated protein kinase
**Max**	MYC associated factor X
**mTORC1**	Mammalian target of rapamycin complex 1
**MZF-2**	Myeloid-specific zinc finger protein 2
**NF-kB**	Nuclear factor kappa B subunit
**NFX1-123**	Nuclear transcription factor, X-box binding 1-123
**NHP2**	NHP2 Ribonucleoprotein
**NOTCH1**	Notch homolog 1
**NOP10**	Nucleolar Protein 10
**NRAS**	Neuroblastoma RAS viral oncogene homolog
**p27KIP1**	Cyclin Dependent Kinase Inhibitor 1B
**p53**	p53 protein
**PIK3CA**	Phosphatidylinositol-4,5-bisphosphate 3-kinase, catalytic subunit alpha
**POL2**	RNA polymerase 2
**POT1**	Protection of telomeres 1
**PRC2**	Polycomb repressor complex 2
**RAP1**	Repressor/activator protein 1
**Snail**	Snail family transcriptional repressor
**Sp1**	Sp1 Transcription Factor
**STAT3**	Signal Transducer and Activator of Transcription 3
**Tax**	HTLV-1 regulatory protein
**TGFβ**	Transforming Growth Factor, Beta
**TERC**	Telomerase RNA component
**TERT**	Telomerase reverse transcriptase
**TIN2**	TRF1 interacting nuclear factor 2
**TPP1**	Tripeptidyl peptidase 1
**TRF1**	Telomeric repeat binding factor 1
**TRF2**	Telomeric repeat binding factor 2
**TRIM24**	Tripartite motif containing 24
**TRIM28**	Tripartite motif containing 28
**WT1**	Wilms tumour-suppressor gene-1
